# Knowledge Assessment of Correct Infant Sleep Practices and Sudden Infant Death Syndrome Among Mothers

**DOI:** 10.7759/cureus.20510

**Published:** 2021-12-19

**Authors:** Abdulrahman F Algwaiz, Ahmed M Almutairi, Abdullah M Alnatheer, Mohammed A Alrubaysh, Osama Alolaiwi, Mohammed Alqahtani

**Affiliations:** 1 Ophthalmology, Imam Mohammed Ibn Saud Islamic University, Riyadh, SAU; 2 College of Medicine, Imam Mohammed Ibn Saud Islamic University, Riyadh, SAU; 3 Pediatrics, Imam Mohammed Ibn Saud Islamic University, Riyadh, SAU; 4 Cardiac Surgery, Imam Mohammed Ibn Saud Islamic University, Riyadh, SAU

**Keywords:** pediatric preventive medicine, parental smoking, health education & awareness, sleep practice, pediatrics & neonatology, sudden infant death syndrome (sids)

## Abstract

Introduction: Sudden infant death syndrome (SIDS) is characterized as the sudden unexpected death of a healthy infant below the age of 12 months with an unknown cause even after careful death scene assessment. The aim of this study is to estimate the percentage of proper sleep practices among infants and assess the knowledge and awareness of SIDS and its associated risk factors among Saudi and non-Saudi mothers.

Methods: This cross-sectional study was done in Riyadh, Saudi Arabia. The data were collected using an anonymous, self-administered questionnaire that consisted of 36 items that were divided into demographic data of the parents and child, observations of the child’s sleep practice, and knowledge and awareness of SIDS and its associated risk factors.

Results: A total of 667 participants completed the questionnaire. The mean age of the mothers was 31.44 + 7.55. As for the nationality, 527 (79%) were Saudi and 140 (21%) were non-Saudi. The majority had bachelors’ degrees 407 (61%). Sleep practices assessment demonstrated that 391 (58.6%) of infants were sleeping in the supine position. A total of 329 (49.3%) participants reported hearing about SIDS from social media and websites as being the major source of information. SIDS acknowledgment was higher in non-Saudi mothers compared to Saudis.

Conclusion: The results provide informative descriptive data on childcare practices in Saudi Arabia. Considerable variation was noted and the results from this study are intended to have a better understanding of the prevalence of childcare practices and knowledge of SIDS risk factors in Saudi and non-Saudi mothers.

## Introduction

Sudden infant death syndrome (SIDS) is defined as the sudden death of a newborn child under one year of age that is surprising and unexplained after a careful postmortem assessment such as assessment of the death scene and autopsy [1,2]. In the United States, it is approximated that 4,000 infants die yearly from sleep-related deaths [3]. Worldwide, the most common causes of infant deaths are birth defects, prematurity, low birth weight, complications during maternal pregnancy, SIDS, and injuries significant enough to cause mortality [4]. Despite the decrease in occurrence during the previous twenty years, SIDS remains the leading cause of infant mortality in the post-neonatal period between one month to one year, with the peak incidence being between two to four months of age [5-7]. Studies found that deaths resulting from SIDS were occurring between midnight and 8:00 AM [4]. SIDS has been long been believed to be multifactorial in origin, with the triple-risk hypothesis proposed by Filiano and Kinney in 1994 being the most widely accepted model. This model proposes that SIDS occurs when three overlapping factors intersect: (I) a vulnerable infant; (II) a critical developmental period for homeostatic control, and (III) an exogenous stressor [8]. SIDS risk factors were divided into modifiable and non-modifiable risk factors. Recognized epidemiological investigations have concurred that a baby’s sleeping position (prone and side), bed-sharing (the most important risk factor in infants younger than four months), soft bedding (e.g., blankets and pillows), unsafe sleep surfaces (e.g., sofas), maternal smoking, and prematurity are the most significant risk factors associated with SIDS [7,9]. While on the other hand, breastfeeding, pacifier use, room-sharing without bed-sharing have shown favorable impacts as protective factors [7,10,11]. In Saudi Arabia, studies on SIDS are limited due to the lack of permission grants for post-mortem examination. This limits medical and epidemiological studies of SIDS in Saudi Arabia. A study that was done in Al-Qatif, Saudi Arabia in 1995 showed that SIDS comprised 6.2% of the infants who died between the age of one and 12 months [12]. Regarding knowledge and awareness of SIDS in Saudi Arabia, the studies are very limited. In this study, we aim first to estimate the percentage of proper sleep practices among infants in Riyadh, Saudi Arabia, and second to assess the knowledge and awareness of SIDS and its associated risk factors among Saudi and non-Saudi mothers.

## Materials and methods

This descriptive, cross-sectional study was carried out in Riyadh, Saudi Arabia. The study was approved by the Institutional Review Board at King Fahad Medical City (21-116E). The period of data gathering was obtained from March 26 to April 12, 2021. The study was performed by an anonymous, self-administered questionnaire that was sent through e-mails and text messages. The subjects were chosen through the hospital registry and clinic visits at King Fahad Medical City and associated primary health care centers. The questionnaire was sent to 800 individuals. A total of 667 replied to us (83.3% response rate). The sample consists of Saudi and non-Saudi mothers with a child who is currently less than a year old and living in Riyadh, Saudi Arabia. Nonprobability convenience sampling was used when selecting the mothers. Our inclusion criteria include mothers with children currently younger than 12 months of age and living in the Riyadh region. We excluded any mother who was not the primary caregiver and children who have clinical reasons for avoiding certain sleep practices (e.g., gastroesophageal reflux disease [GERD], congenital upper airway malformation). The self-administered questionnaire includes 36 items that were written and reviewed by three independent pediatric consultants, with one of them being a neonatal intensive care unit specialist. A pilot study was performed for validation before the initiation of the study. The questionnaire is divided into three parts. The first part includes items for collecting demographic data of the parents and the child, the second part included observations of the child’s sleep practices, and finally, the third part included items to assess the knowledge and awareness of SIDS and its associated risk factors. Information was gathered in a confidential manner and the study protocol was approved by a local human ethics committee at King Fahad medical city. Data were entered in Microsoft Excel 2016 and analyzed using IBM SPSS (statistical package of social science) rendition 24 (IBM Corp., Armonk, NY, USA) for investigation. Frequencies and percentages were used to present categorical variables and mean and standard deviation for numerical variables. The chi-squared test is used for comparison of the level of the mothers’ SIDS knowledge and proper sleep practices. Any test declared significant at a p-value < 0.05. The confidence interval (CI) of 95% while keeping in consideration the margin of error being 5%.

## Results

Socio-demographic information of the participants

Table 1 shows the socio-demographic profile of the participants. The mean age of mothers was 31.44 + 7.55. As for the nationality, 527 (79%) were Saudi and 140 (21%) were non-Saudi. As for the city, 598 (89.7%) were living in urban areas (in Riyadh city) and 69 (10.3%) were living in a rural area (in the Riyadh region). Regarding the education level of the mothers, the majority had bachelors’ degrees 407 (61%). The same applies to the education level of the fathers which was 648 (52.2%) for bachelor’s degrees. As for the mothers’ occupational status, 287 (43%) were working, while 380 (57%) were housewives. As for the smoking status of the families, 176 (26.4%) had a smoking father, nine (1.3%) had a smoking mother, 19 (2.8%) had both the parents smoking, and 463 (69.4%) had none of the parents smoking. The mean age of the infants in months was 6.48 + 3.28. As for the infants’ gender, 286 (42.9%) were males, and 381 (57.1%) were females. For 294 (44.1%), the participating infant was their first and 27 (4%) of the infants were born premature or with low birth weight. As for what kind of milk the mothers were planning to feed their infants during the first two months, 224 (33.6%) stated breastfeeding only, 77 (11.5%) formula feeding only, 307 (46%) combined feeding with continued breastfeeding for the first two months, and 59 (8.8%) combined feeding without continued breastfeeding for two months.

**Table 1:** Socio-demographic profile of the participants (n = 667)

Demographical characteristic		n	%
Relationship to the child			
Mother		667	100
Age of the participants			
Mean		31.44	
Standard deviation		7.55	
Nationality			
Saudi		527	79
Non-Saudi		140	21
City			
Urban		598	89.70
Rural		69	10.30
Education level of mother			
Primary school		9	1.30
Elementary school		15	2.20
High school		119	17.80
Bachelor's degree		407	61.00
Master/PhD		111	16.60
None of the above		6	0.90
Education level of father			
Primary school		4	0.60
Elementary school		19	2.80
High school		119	17.80
Bachelor's degree		348	52.20
Master/PhD		169	25.30
None of the above		8	1.20
Mother occupation status			
Working		287	43.00
Housewife		380	57.00
Income			
Less than 10,000 SR		210	31.50
Between 10,000 and 20,000 SR		301	45.10
More than 20,000 SR		156	23.40
Does any of the parents' smoke?			
Father		176	26.40
Mother		9	1.30
Both		19	2.80
None		463	69.40
Age of the infants in months			
Mean		6.48	
Standard deviation		3.28	
Infant gender			
Male		286	42.9
Female		381	57.1
Is this your first child			
Yes		294	44.10
No		373	55.90
Was your child born preterm (<37 weeks) or with low birth weight (<2.5 kg)?			
Yes		27	4.00
No		549	82.30
I do not know		91	13.60
What is the feeding method you’re using or intend to follow during the first two months of your baby's life?			
Breastfeeding only		224	33.60
Formula feeding only		77	11.50
Combine feeding with continued breastfeeding for two months		307	46.00
Combine feeding without continued breastfeeding for two months		59	8.80

Assessing Sleep Practices

Table 2 demonstrates the sleep practice of the participating infants. Eighty three (12.4%) of infants were sleeping in the prone position, 391 (58.6%) were sleeping in the supine position, and 193 (28.9%) were sleeping in the side position. Fifty five (8.2%) stated that the infant at least slept once in a separate room from the caregiver before the age of six months, 93 (13.9%) with the infant at least slept once in a separate room from the caregiver before the age of six months, and 519 (77.9%) where the infant never slept in a separate room. Sixteen (10.81%) of mothers with infants that slept in a separate room mentioned that this has occurred due to exceptional circumstances, 280 (42%) reported the infant co-slept with a parent before the age of four months, and 387 (58%) occurred after the age of four months. A total of 124 (18.6%) of infants co-slept with a person other than the parents on the same bed and 92 (13.8%) infants co-slept with a smoker parent in the same bed.

**Table 2:** Sleep practice of participants' children (n = 667)

Question		n	%
Q1/ How does the infant usually sleep?			
Prone		83	12.4
Supine		391	58.6
Side		193	28.9
Q2/ Do you use a sleeping sack?			
Yes		120	18
No		547	82
Q3/ Do you put a pillow inside the baby’s crib?			
Yes		438	65.7
No		229	34.3
Q4/ Do you use a cot buffer?			
Yes		520	78
No		147	22
Q5/ Do you use a soft mattress?			
Yes		610	91.5
No		57	8.5
Q6/ Do you use a plastic mattress cover?			
Yes		432	64.8
No		235	35.2
Q7/ Do you turn on air-conditioning (cold setting) when the child is sleeping in the summer?			
Yes		491	73.6
No		176	26.4
Q8/ Do you turn on air-conditioning (hot setting) when the child is sleeping in the winter?			
Yes		176	26.4
No		491	73.6
Q9/ Does the infant use a pacifier when he is sleeping?			
Yes		204	30.6
No		463	69.4
Q10/ Is the infant swaddled in general?			
Yes		313	46.9
No		354	53.1
Q11/ Does the infant have a soft toy in his crib while he is sleeping?			
Yes		422	63.3
No		245	36.7
Q12/ Has the infant ever slept in a separate room from the parents or a caregiver?			
Yes, it occurred before the age of 6 months		55	8.2
Yes, it occurred after the age of 6 months		93	13.9
No		519	77.8
Q13/ If the answer to the previous question was yes, did it occur because of an exceptional circumstance?			
Yes		16	10.81
No		132	89.19
Q14/ Has the infant ever co-slept with the parents in the same bed?			
Yes, it occurred before the age of 4 months		280	42
Yes, it occurred after the age of 4 months		387	58
Q15/ Has the infant ever co-slept with a person (other than the parents) in the same bed?			
Yes		124	18.6
No		543	81.4
Q16/ Has the infant ever co-slept with a smoker parent in the same bed?			
Yes		92	13.8
No		575	86.2

Bedsharing Habits

Around 400 (60%) participating mothers had unsafe bedsharing habits (defined by bedsharing infants younger than four months, or sharing a bed with a smoker parent or sharing a bed with a premature infant), and 267 (40%) reported safe bedsharing habits (defined as bedsharing for infants older than four months, in full-term with normal birth weight infants who shared a bed with both parents being non-smokers).

*Sleeping Position* 

**Figure 1:**
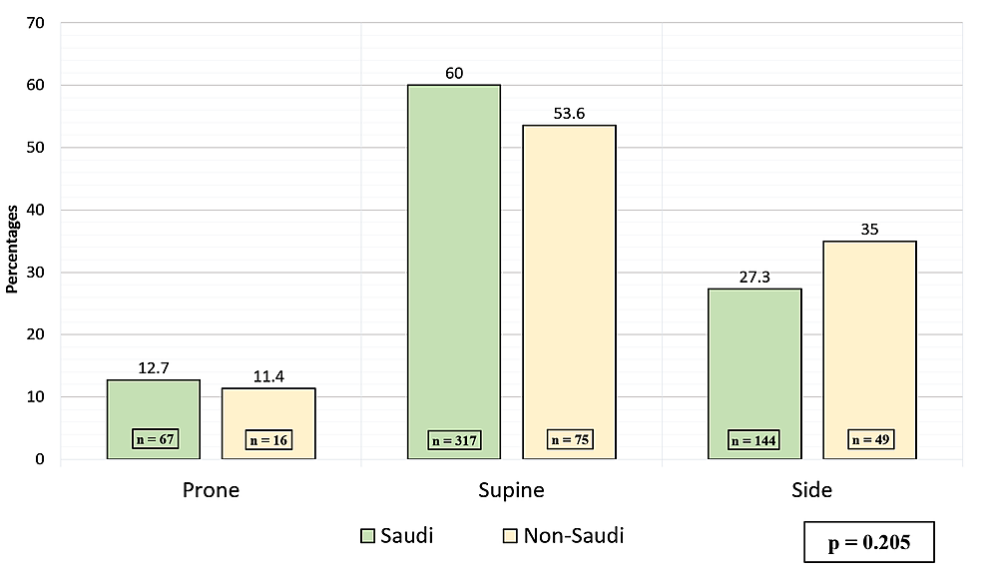
Sleeping position across nationality

Figure 1 illustrates the infants sleeping position across nationalities No significant difference was found between Saudi and non-Saudi infants in the sleeping position. Similar trends of sleeping positions were observed across Saudis and non-Saudis.

SIDS awareness and source of knowledge

**Figure 2:**
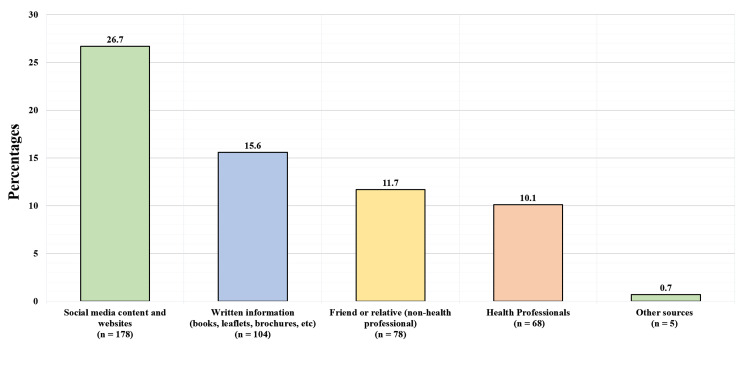
Source of knowledge toward SIDS in participants who heard about it before

A total of 329 (49.3%) participants have heard about SIDS before, and 338 (50.7%) have never heard about it before. Figure 2 displays the source of knowledge toward SIDS among participants who reported hearing about it before. One hundred seventy eight (26.7%) from social media and websites, 104 (15.6%) written information (books, brochures), 78 (11.7%) friends and families (non-health professionals), 68 (10.1%) health professionals, and five (0.7%) had other sources.

SIDS risk factors knowledge assessment

Table 3 demonstrates the knowledge assessment toward risk factors of SIDS among participants who previously heard about it. The mean knowledge score was 3.32 + 1.62, the minimum was 0, and the maximum was 6. As for the knowledge classification, 161 (48.9%) were not aware, since their score was half and lower (3 or less), 144 (43.8%) had acceptable awareness (had a score between 4 and 5), and 24 (7.3%) were fully aware (had a score of 6).

**Table 3:** Knowledge assessment toward sudden infantile death syndrome (SIDS) (n = 329)

Question		n	%
Which of the following do you think is a risk factor for SIDS?			
1/ Sleeping position other than supine:			
Yes		179	54.4
No		60	18.2
I do not know		90	27.4
2/ Soft objects and loose bedding:			
Yes		158	48
No		84	25.5
I do not know		87	26.4
3/ Using a pacifier at nap time and bedtime:			
Yes		109	33.1
No		143	43.5
I do not know		77	23.4
4/ Overheating and head covering:			
Yes		216	65.7
No		51	15.5
I do not know		62	18.8
5/ Sharing the bed with the infant:			
Yes		216	65.7
No		56	17
I do not know		57	17.3
6/ Smoke exposure during pregnancy and after birth			
Yes		181	55
No		62	18.8
I do not know		86	26.1
Knowledge score (Highest possible score = 6, lowest possible score = 0)			
Mean			3.32
Standard deviation			1.62
Minimum			0
Maximum			6
Knowledge classification			
Knowledge class		n	%
Not aware (score of 3 or less)		161	48.90
Accepted awareness (score between 4 and 5)		144	43.80
Fully aware (score of 6)		24	7.30

Factors Associated With the Participants Who Previously Heard of SIDS

Table 4 shows the factors associated with previously hearing about SIDS. Nationality was significantly associated with previously knowing about SIDS (p = 0.002), whereas a higher rate of non-Saudis knew about SIDS compared to Saudis (60.7% vs 46.3%). Having a child born premature or with low birth weight was also significantly associated with previously knowing about SIDS (p < 0.001), whereas those who did not if their child was premature/had low birth weight had a notable lower rate of knowing about SIDS (29.7%) compared to those who had a child who was premature/had low birth weight (55.6%) and those who did not have a child who was born premature/had low birth weight (52.3%). Mothers’ age, infants’ age, city, mothers’ education, fathers’ education, mothers’ occupation status, income, smoking status, infant gender, and having a first child were not significantly associated with previously hearing about SIDS.

**Table 4:** Factors associated with previously knowing about sudden infantile death syndrome (SIDS) *Significant at level 0.05

Factor		Have you ever heard about sudden infantile death syndrome (SIDS)?		P-value
		Yes	No	
Mother age (mean, SD)		31.18 + 7.12	31.69 + 7.95	0.053
Child age in months (mean, SD)		6.69 + 3.47	6.28 + 3.09	0.106
Nationality				0.002*
Saudi		244 (46.3%)	283 (53.7%)	
Non-Saudi		85 (60.7%)	55 (39.3%)	
City				0.451
Urban		292 (48.8%)	306 (51.2%)	
Rural		37 (53.6%)	23 (46.4%)	
Education level of mother				0.183
Primary school		6 (66.7%)	3 (33.3%)	
Elementary school		3 (20%)	12 (80%)	
High school		54 (45.4%)	65 (54.6%)	
Bachelor's degree		205 (50.4%)	202 (49.6%)	
Master/PhD		58 (52.3%)	53 (47.7%)	
None of the above		3 (50%)	3 (50%)	
Education level of father				0.668
Primary school		2 (50%)	2 (50%)	
Elementary school		7 (36.8%)	12 (63.2%)	
High school		61 (51.3%)	58 (48.7%)	
Bachelor's degree		177 (50.9%)	171 (49.1%)	
Master/PhD		77 (45.6%)	92 (54.4%)	
None of the above		5 (62.5%)	3 (37.5%)	
Mother occupation status				0.475
Working		137 (47.7%)	150 (52.3%)	
Housewife		192 (50.5%)	188 (49.5%)	
Income				0.237
Less than 10,000 SR		113 (53.8%)	97 (46.2%)	
Between 10,000 and 20,000 SR		139 (46.2%)	162 (53.8%)	
More than 20,000 SR		77 (49.4%)	79 (50.6%)	
Does any of the parents' smoke?				0.103
Father		84 (47.7%)	92 (52.3%)	
Mother		1 (11.1%)	8 (88.9%)	
Both		11 (57.9%)	8 (42.1%)	
None		233 (50.3%)	230 (49.7%)	
Child gender				0.631
Male		138 (48.3%)	148 (51.7%)	
Female		191 (50.1%)	190 (49.9%)	
Is this your first child?				0.998
Yes		145 (49.3%)	149 (50.7%)	
No		184 (49.3%)	189 (50.7%)	
Was your child born preterm (<37 weeks) or with low birth weight (< 2.5 kg)?				< 0.001*
Yes		15 (55.6%)	12 (44.4%)	
No		287 (52.3%)	262 (47.7%)	
I do not know		27 (29.7%)	64 (70.3%)	

*Factors Associated With the Knowledge Level in Participants Who Previously Heard of *​​​*SIDS* 

Table 5 displays the factors associated with knowledge level toward SIDS among participants who previously heard about it. Mothers’ education was significantly associated with their level of knowledge (p = 0.006), whereas it was observed that the higher the mothers' education, the higher the level of knowledge. Mothers’ occupation status was also significantly associated with knowledge level (p = 0.045), whereas working mothers had notably higher knowledge levels compared to housewives. Having a child born premature or with low birth weight was also significantly associated with knowledge (p = 0.011), where it was seen that mothers who did not have a premature child or child with low birth weight were seen to have higher knowledge level compared to those who had and those who did not know. Mothers’ age, infants’ age, nationality city, fathers’ education, income, smoking status, Infant gender, having a first child and were not significantly associated with knowledge level toward SIDS.

**Table 5:** Factors associated with knowledge level toward sudden infantile death syndrome (SIDS) *Significant at level 0.05

Factor		Knowledge level			P-value
		Not aware	Accepted awareness	Fully aware	
Mother age (mean, SD)		31.36 + 7.47	31.31 + 6.65	29.21 + 5.13	0.371
Child age in months (mean, SD)		6.61 + 3.44	6.73 + 3.52	6.92 + 3.52	0.907
Nationality					0.223
Saudi		125 (51.2%)	100 (41%)	19 (7.8%)	
Non-Saudi		36 (42.4%)	44 (51.8%)	5 (5.9%)	
City					0.395
Urban		139 (47.6%)	131 (44.9%)	22 (7.5%)	
Rural		22 (59.5%)	13 (35.1%)	2 (5.4%)	
Education level of mother					0.006*
Primary school		6 (100%)	0 (0%)	0 (0%)	
Elementary school		3 (100%)	0 (0%)	0 (0%)	
High school		32 (59.3%)	21 (38.9%)	1 (1.9%)	
Bachelor's degree		91 (44.4%)	101 (49.3%)	13 (6.3%)	
Master/PhD		27 (46.6%)	21 (36.2%)	10 (17.2%)	
None of the above		2 (66.7%)	1 (33.3%)	0 (0%)	
Education level of father					0.151
Primary school		0 (0%)	2 (100%)	0 (0%)	
Elementary school		6 (85.7%)	1 (14.3%)	0 (0%)	
High school		30 (49.2%)	27 (44.3%)	4 (6.6%)	
Bachelor's degree		78 (44.1%)	87 (49.2%)	12 (6.8%)	
Master/PhD		45 (58.4%)	24 (31.2%)	8 (10.4%)	
None of the above		2 (40%)	3 (60%)	0 (0%)	
Mother occupation status					0.045*
Working		59 (43.1%)	63 (46%)	15 (10.9%)	
Housewife		102 (53.1%)	81 (42.2%)	9 (4.7%)	
Income					0.535
Less than 10,000 SR		54 (47.8%)	52 (46%)	7 (5.2%)	
Between 10,000 and 20,000 SR		71 (51.1%)	60 (43.2%)	8 (5.8%)	
More than 20,000 SR		36 (46.8%)	32 (41.6%)	9 (11.7%)	
Does any of the parents' smoke?					0.723
Father		41 (48.8%)	39 (46.4%)	4 (4.8%)	
Mother		1 (100%)	0 (0%)	0 (0%)	
Both		5 (45.5%)	6 (54.5%)	0 (0%)	
None		114 (48.9%)	99 (42.5%)	20 (8.6%)	
Child gender					0.727
Male		71 (51.4%)	57 (41.3%)	10 (7.2%)	
Female		90 (47.1%)	87 (45.5%)	14 (7.3%)	
Is this your first child?					0.140
Yes		77 (53.1%)	55 (37.9%)	13 (9%)	
No		84 (45.7%)	89 (48.4%)	11 (6%)	
Was your child born preterm (<37 weeks) or with low birth weight (< 2.5 kg)?					0.011*
Yes		11 (73.3%)	4 (26.7%)	0 (0%)	
No		130 (45.3%)	133 (46.3%)	24 (8.4%)	
I do not know		20 (74.1%)	7 (25.9%)	0 (0%)	

## Discussion

Multiple campaigns, including the “back to sleep” campaign, during which parents were advised to avoid the prone sleeping position, overheating, swaddling, and parental smoking, resulted in a significant fall in SIDS rates in all western countries that undertook these campaigns [13]. In Ireland, SIDS rates fell from 2.1 out of 1,000 live births in 1980-1990 to 0.7-0.8 for the years 1994-2000, which indicates 70 to 80 fewer infants dying in a year [14]. In Japan, a study that involved 4,319 parents of newborns showed that almost all parents (96.7 %) avoid laying infants down in the prone position [3]. While nearly all parents chose exclusive supine positioning, only 81.4%% of parents were aware that the prone position was considered as a risk factor for SIDS. While in Turkey, putting the child to sleep in the supine position was practiced by 46.7% of families [2]. There are limited data about SIDS from developing countries [15-18]. In the United Arab Emirates (UAE), 72.2% of mothers preferred the supine position compared to other positions when putting their infants to bed [14]. SIDS prevalence in Saudi infants is still unknown. The supine sleeping position (i.e., the only correct sleeping position) was practiced by 60% and 53.6% Saudi and non-Saudi mothers, respectively. The prone sleeping position, which has consistently been shown to increase the risk of SIDS in infants [8], was 12.7% and 11.4% among Saudi and Non-Saudi, respectively. These results are higher than what was observed in other countries in Asia, northern Europe, and New Zealand. but still lower than those in the United States of America and Southern Europe [19]. In previous literature, placing infants on their sides was initially considered safe as placing them supine, but later, studies showed infants were twice as likely to die from SIDS if they were placed on their sides [20]. Approximately 27.3% and 35% of infants to Saudi and non-Saudi mothers in the present study slept on their sides at one time or another, thus exposing them to a greater risk of SIDS. We found no correlation between the different sleeping positions and the mothers’ nationality, income, and education level. Bedsharing has been implicated as a risk factor for SIDS. In the past, infants sharing the bed with parents or caregivers who smoked demonstrated an increased risk for SIDS [21,22]. However, there have been many studies suggesting that bedsharing is a risk factor on its own, even without the paternal or maternal smoking role [23]. Nonetheless, maternal smoking is considered one of the most important risk factors for SIDS [24]. As shown in our study, 1% of mothers and 26.4% of fathers were smokers. No epidemiologic studies have proposed a protective effect from bedsharing; hence bedsharing should not be encouraged as a method of reducing SIDS risk. In Turkey, bedsharing was reported in 16% of the parents [2]. In the present study, 60% had unsafe bedsharing habits, which is defined as bedsharing infants younger than four months, sharing a bed with a smoker parent, or sharing a bed with a premature infant. 18.6% out of the 60% respondents stated that the infant co-slept with a smoker parent in the same bed. As for room sharing without bedsharing, it was found to reduce the risk of SIDS and remove the possibility of suffocation, strangulation, and entrapment that may occur when the infant is sleeping in the adult bed, especially during the first six months [25]. In the present study, 77.8% of mothers stated their infant has never slept in a separate room from the parents or a caregiver. Soft mattresses, pillows, and cot buffers have been associated with a 2-to-3-fold increased risk of SIDS [26]. An even greater risk results from combining multiple risk factors exist, for example, sleeping in the prone position in soft bedding has been associated with a 20-fold increased risk of SIDS [27]. In this study, 65% of mothers used pillows, 91.5% used soft mattresses, and 78% used cot buffers for the infant’s crib. 46.9% of mothers swaddled their infants most of the time. Overheating as a result of increased room temperature, high body temperature, sweating, and excessive clothing or bedding has been associated with an increased risk of SIDS [15]. Multiple studies have identified an interaction between overheating and sleeping in the prone position, with overheating increasing the risk of SIDS 6-to-10 fold only among infants sleeping in the prone position [25,28,29]. In the UAE, more than 80% of families had used childcare practices that can result in overheating (e.g., bedding duvet in the summer, increased room temperature, and excessive clothing) [14]. Although the mechanism of protection is yet unclear, studies have reported a protective effect of pacifiers on the incidence of SIDS. The protective effect of the pacifier is observed even if the pacifier falls out of the infant’s mouth [30-33]. In the present study, 30.6% reported using a pacifier for their infants during sleeping. Breastfeeding has been proven to be protective against SIDS, and this effect is stronger when breastfeeding is exclusive [11]. Unless contraindicated, mothers should exclusively breastfeed or feed with expressed milk (i.e., not offer any formula or other nonhuman milk-based supplements) for six months [34]. We found that only 33.6% of participating mothers stated to only breastfeed during the first two months, 11.5% for formula feeding only, and 46% combined with continued breastfeeding for the first two months. Worldwide, the knowledge and awareness of proper sleep practices and other SIDS-associated risk factors are variable. In France, a study was done on 202 pregnant women that displayed 94.6% of women stated that they had heard about SIDS before. In Saudi Arabia, there were no campaigns done to educate parents about correct sleeping practices and avoiding other SIDS risk factors. Nonetheless, almost half of the mothers (49.3%) reported having heard of SIDS before. When assessing the level of awareness of SIDS, the results were poor with 7.3% being fully aware and 43.8% having acceptable awareness. The awareness of SIDS among non-Saudi mothers was higher than Saudi mothers (60.7%% vs 46.3%). In France, a study on 202 pregnant women displayed 94.6% of women stated that they had heard about SIDS before [13]. This percentage of awareness is almost double the percentage of Saudi mothers who have heard about SIDS in our report. In addition, the present study shows the maternal awareness of SIDS among residents was higher than our citizens. This knowledge gap among Saudis highlights the importance of implementing educational interventions and campaigns to improve public awareness regarding the correct sleeping practices and avoiding other SIDS risk factors. The majority of our participating mothers reported that media platforms (social media and websites) were the main source of information from where they heard about SIDS, which was found to be similar to other previous studies done in France and Turkey [2,13]. This study suggests that families might be informed effectively about SIDS by way of the media. Education campaigns to the public promoting supine sleeping positions and discouraging other unsafe childcare practices may help to educate mothers in Saudi Arabia further. Our main limitation was the method sampling and selection bias which was conventional. We assessed the role of maternal education level as a risk factor or potential confounder, but it proved not to be a risk factor for placing infants in a prone position.

## Conclusions

To conclude, these results provide informative descriptive data on childcare practices in Saudi Arabia and are the first of such work on infants in the Middle East. Considerable variation was noted in all the practices described. The results from this study are not intended to be used to imply that any particular childcare practice either has a role in increasing or decreasing the risk of SIDS, but instead to better understand the prevalence of childcare practices and knowledge of SIDS risk factors in Saudi and non-Saudi mothers in Saudi Arabia. These data provide useful baseline information and should be of great benefit to the health authorities should they choose to develop strategies to reduce the risk of SIDS, especially among preterm infants, since they have a higher risk of SIDS.

## Appendices

Questionnaire (English)

Epidemiology of the study population:

A/ Parent’s data

1-What is your relationship to the child:

A.             Mother

B.             Father

C.             Other

*Since the study targets mothers, if a father or “other” were chosen, the questionnaire will end.

2-Mother’s age? (in years)

3-What is your nationality?

A.             Saudi

B.             Non-Saudi

4-Where do you live?

A.             In urban area (city)

B.             In rural areas

5-What is the highest education level for the child's mother?

A.     Primary school

B.      Intermediate school

C.      Secondary school

D.     University or college

E.      Postgraduate

F.      None of the above

6-What is the highest education level for the child's father?

A.     Primary school

B.      Intermediate school

C.      Secondary school

D.     University or college

E.      Postgraduate

F.      None of the above

7-Mother’s Occupation:

A.             Working

B.             Not working (Housewife)

8-What is the average household income (per month)?

A.             Less than 10,000 Saudi Riyal

B.             10,000-20,000 Saudi Riyal

C.             More than 20,000 Saudi Riyal

9-Do any of the parents smoke?

A.        Father

B.        Mother

C.        Both

D.        None

B/ Child data

1-What is the age of the infant (in months)?

____ months

2- Child’s gender:

A.      Male

B.      Female

3-Is this your first child:

A.             Yes

B.             No

4- Was your child born preterm (<37 weeks) or with low birth weight (<2.5kg)?

A.             Yes

B.             No

C.        I don’t know

5- Does the infant have a birth defect in his upper respiratory system and/or is taking medications for Gastroesophageal Reflux Disease (GERD)

A.             Yes

B.             No

C.        I don’t know

6- What kind of milk did you use or plan to use for your infant during the first two months of life?

A.         Mother’s breast milk only

B.         Formula milk only

C.        Both

7- What is the feeding method you’re using or intend to follow during the first two months of your baby's life?

A.        Breastfeeding only

B.         Formula feeding only

C.        Combine feeding with continued breastfeeding for two months

D.        Combine feeding without continued breastfeeding for two months

Sleep practices:

All questions are targeted towards the child

1-How does the infant usually sleep?

A.            Prone

C.            Supine

D.            Side

2- Do you use a bedding Duvet during summer?

A.             Yes

B.             No

3- Do you use a bedding Duvet during winter?

A.             Yes

B.             No

4- Do you use a sleeping sack? (Example picture)

A.             Yes

B.             No

5- Do you put a pillow inside the baby’s crib?

A.             Yes

B.             No

6- Do you use a cot buffer? (Example picture)

A.             Yes

B.             No

7- Do you use a soft mattress?

A.             Yes

B.             No

8- Do you use a plastic mattress cover? (Example picture)

A.             Yes

B.             No

9- Do you turn on air-conditioning (cold setting) when the child is sleeping in the summer?

A.             Yes

B.             No

10- Do you turn on air-conditioning (hot setting) when the child is sleeping in the winter?

A.             Yes

B.             No

11- Does the infant use a pacifier when he is sleeping?

A.             Yes

B.             No

12- Is the infant swaddled in general? (Example picture)

A.             Yes

B.             No

13- Does the infant have a soft toy in his crib while he is sleeping?

A.             Yes

B.             No

14- Has the infant ever slept in a separate room from the parents or a caregiver?

A.     Yes, it occurred before 4 months of age

B.      Yes, it occurred after 4 months of age

C.      No

15- If the answer to the previous question was yes, did it occur because of an exceptional circumstance?

A.     Yes (mention)

B.      No

16- Has the infant ever co-slept with the parents in the same bed?

D.     Yes, it occurred before 4 months of age

E.      Yes, it occurred after 4 months of age

F.      No

17- Has the infant ever co-slept with a person (other than the parents) in the same bed?

A.     Yes

B.      No

18- Has the infant ever co-slept with a smoker in the same bed?

A.     Yes

B.      No

Questions about SIDS:

1- Have you ever heard about Sudden Infantile Death Syndrome (SIDS)?

A.            Yes

B.             No

2- If the answer to the previous question was yes, from where did you hear it? (multiple choice)

A.    My child’s physician

B.     Friend or relative (non-health professional)

C.     Friend or relative (health professional)

D.    Written information (for example, books, leaflets, brochures, etc)

E.     Social media content and websites

F.      Other

Which of the following do you think is a risk factor for SIDS?

Q1/ Sleeping position other than supine:

●       Yes

●       No

●       I don’t know

Q2/ Soft objects and loose bedding:

●       Yes

●       No

●       I don’t know

Q3/ Using a pacifier at nap time and bedtime:

●       Yes

●       No

●       I don’t know

Q4/ Overheating and head covering:

●       Yes

●       No

●       I don’t know

Q5/ Sharing the bed with the infant:

●       Yes

●       No

●       I don’t know

Q6/ Smoke exposure during pregnancy and after birth

●       Yes

●       No

●       I don’t know
